# Supplementary material for: From sequence to enzyme mechanism using multi-label machine learning
Source: BMC Bioinformatics. 2014 May 19;15:150. doi: 10.1186/1471-2105-15-150 (PMC4229970; doi:10.1186/1471-2105-15-150)
Supplement: Additional file 2 — Java code of ml2db. Additional file ml2db_code.tar.gz contains the Java source code to run the multi-label machine learning experiments and save the results to database. The code’s Javadoc is included. [file 1471-2105-15-150-S2.zip › additional file 2/ml2db/utils/doc/uk/ac/ed/inf/utils/maputils/OneToManyMap.html]

OneToManyMap


---


|  |  |  |
| --- | --- | --- |
| **Overview** \| **Package** \| **Class** \| **Use** \| **Tree** \| **Deprecated** \| **Index** \| **Help** | |  |
| **PREV CLASS**   **NEXT CLASS** | **FRAMES**    **NO FRAMES**     **All Classes** |  |
| SUMMARY: NESTED \| FIELD \| CONSTR \| METHOD | DETAIL: FIELD \| CONSTR \| METHOD |  |


---


## uk.ac.ed.inf.utils.maputils Class OneToManyMap<T,U>

```
java.lang.Object
  java.util.AbstractMap<K,V>
      java.util.TreeMap<T,java.util.Set<U>>
          uk.ac.ed.inf.utils.maputils.OneToManyMap<T,U>
```

**All Implemented Interfaces:**: java.io.Serializable, java.lang.Cloneable, java.util.Map<T,java.util.Set<U>>, java.util.NavigableMap<T,java.util.Set<U>>, java.util.SortedMap<T,java.util.Set<U>>

**Direct Known Subclasses:**: IndexedOneToManyMap

---

``` public class OneToManyMap<T,U> extends java.util.TreeMap<T,java.util.Set<U>> ```

A map containing for each key a set of values. No key can be null. At most
one key can be an empty-string. No value can be null (the Java TreeSet class
used uses "null" as a return message when there is \*no\* mapping for a given
key!). A value can be an empty string.

**Version:**
:   3 Jun 2008

**Author:**
:   Luna De Ferrari luna.deferrari-at-ed.ac.uk

**See Also:**: Serialized Form

---

**Nested Class Summary**

**Nested classes/interfaces inherited from class java.util.AbstractMap**

`java.util.AbstractMap.SimpleEntry<K,V>, java.util.AbstractMap.SimpleImmutableEntry<K,V>`


**Constructor Summary**

`OneToManyMap()`
  
          Initialise an empty map

`OneToManyMap(java.util.Vector<T> keys,
java.util.Vector<U> values)`
  
          Initialise with a list of key-value pairs


| **Method Summary** | |
| --- | --- |
| `void` | `addAll(java.util.Map<T,U> map)` |
| `void` | `addAll(OneToManyMap<T,U> mapToAdd)`             Add all elements of a one2many map to another map |
| `boolean` | `addKeyAndValue(T key, U value)`             Add a key-value pair to the map |
| `void` | `clear()` |
| `boolean` | `containsKey(java.lang.Object key)`             Checks if the map contains a key |
| `boolean` | `containsKeyValue(T key, U value)`             Checks if the map contains a specific key-value pair |
| `boolean` | `containsValue(java.lang.Object value)`             Checks if the map contains a value |
| `boolean` | `deleteKeyValue(T key, U value)` |
| `java.util.Set<U>` | `generateAKeyValues(T key)` |
| `java.util.Set<U>` | `getAKeyValues(T key)`             Get the set of unique values for this key. |
| `java.util.Vector<java.util.Vector>` | `getKeysVectorValuesVector()`             Get 1. |
| `java.util.TreeSet<T>` | `getKeysWithValue(U value)`             Returns all the keys having a certain value |
| `int` | `getKeyValuesPairsSize()`             Get the number of unique key-value pairs in the map. |
| `java.util.Set<T>` | `getNonEmptyKeys()`             Get the set of unique, non-empty-string keys for this map. |
| `int` | `getNonEmptyKeyValuesPairsSize()`             Get the number of unique, non-empty key-value pairs in the map. |
| `java.util.Set<U>` | `getNonEmptyValues()`             Get the set of unique, non-empty-string values for this map. |
| `U` | `getRandomValue(T key)`             Get randomly one of the values of this key |
| `java.util.Set<U>` | `getValues()`             Get the set of unique values for this map. |
| `static OneToManyMap<java.lang.String,java.lang.String>` | `invertKeysAndValues(OneToManyMap<java.lang.String,java.lang.String> map)` |
| `boolean` | `isEmpty()` |
| `boolean` | `mapIsNullOrEmpty()`             public void initialise(Vector keys, Vector values) { this.setMap(); this.generateMap(keys, values); } |
| `boolean` | `removeKey(T key)` |
| `void` | `removeValue(T key, U value)`             Remove a value from the key's list of values |
| `void` | `removeValue(U value)`             Remove a value from \*all\* the key's list of values |
| `void` | `removeValues(java.util.Collection<U> values)` |
| `java.lang.String` | `toCSV()`             Get a list of comma separated [key, value] couples. |
| `java.lang.String` | `toString()` |

**Methods inherited from class java.util.TreeMap**

`ceilingEntry, ceilingKey, clone, comparator, descendingKeySet, descendingMap, entrySet, firstEntry, firstKey, floorEntry, floorKey, get, headMap, headMap, higherEntry, higherKey, keySet, lastEntry, lastKey, lowerEntry, lowerKey, navigableKeySet, pollFirstEntry, pollLastEntry, put, putAll, remove, size, subMap, subMap, tailMap, tailMap, values`

**Methods inherited from class java.util.AbstractMap**

`equals, hashCode`

**Methods inherited from class java.lang.Object**

`getClass, notify, notifyAll, wait, wait, wait`

**Methods inherited from interface java.util.Map**

`equals, hashCode`

**Constructor Detail**

### OneToManyMap

```
public OneToManyMap()
```

:   Initialise an empty map

---


### OneToManyMap

```
public OneToManyMap(java.util.Vector<T> keys,
                    java.util.Vector<U> values)
```

:   Initialise with a list of key-value pairs

    **Parameters:**: `keys` - the list of keys: `values` - the list of values (the value for each key in the keys list)


**Method Detail**

### addAll

```
public void addAll(OneToManyMap<T,U> mapToAdd)
```

:   Add all elements of a one2many map to another map

    :   **Parameters:**: `map` - the map whose elements have to be added

---


### addKeyAndValue

```
public boolean addKeyAndValue(T key,
                              U value)
```

:   Add a key-value pair to the map

    :   **Parameters:**: `key` - a key: `value` - a value **Returns:**: true if the value was added, false if the key-value pair already existed

---


### containsKey

```
public boolean containsKey(java.lang.Object key)
```

:   Checks if the map contains a key

    :   **Specified by:**: `containsKey` in interface `java.util.Map<T,java.util.Set<U>>` **Overrides:**: `containsKey` in class `java.util.TreeMap<T,java.util.Set<U>>`
    :   **Parameters:**: `key` - **Returns:**: true if the key exists in the map, false otherwise

---


### containsKeyValue

```
public boolean containsKeyValue(T key,
                                U value)
```

:   Checks if the map contains a specific key-value pair

    :   **Parameters:**: `key` -: `value` - **Returns:**: true if the key-value exists, false otherwise (false if the key or value are null)

---


### containsValue

```
public boolean containsValue(java.lang.Object value)
```

:   Checks if the map contains a value

    :   **Specified by:**: `containsValue` in interface `java.util.Map<T,java.util.Set<U>>` **Overrides:**: `containsValue` in class `java.util.TreeMap<T,java.util.Set<U>>`
    :   **Parameters:**: `value` - a value **Returns:**: true if the value exists in the map, false otherwise

---


### getKeysWithValue

```
public java.util.TreeSet<T> getKeysWithValue(U value)
```

:   Returns all the keys having a certain value

    :   **Parameters:**: `value` - a value **Returns:**: the list of keys having that value or null if no keys with that value were found

---


### deleteKeyValue

```
public boolean deleteKeyValue(T key,
                              U value)
```

---


### removeKey

```
public boolean removeKey(T key)
```

---


### getRandomValue

```
public U getRandomValue(T key)
```

:   Get randomly one of the values of this key

    :   **Parameters:**: `key` -

---


### getAKeyValues

```
public java.util.Set<U> getAKeyValues(T key)
```

:   Get the set of unique values for this key. No values can be null. At most
    one of the values can be an empty string.

    :   **Returns:**: the set of unique values for this key

---


### generateAKeyValues

```
public java.util.Set<U> generateAKeyValues(T key)
```

---


### getKeysVectorValuesVector

```
public java.util.Vector<java.util.Vector> getKeysVectorValuesVector()
```

:   Get 1. a first vector containing each key as many times as values exist
    for the key. 2. a second vector containing one corresponding value for
    each key entry in the first vector. The two vectors together create a 2
    vectors representation of the map (key in vector 1, value in vector 2).
    Example, for a map: [1:{a, b}, 2:{b, c}] the keys vector will contain:
    [1, 1, 2, 2]. While the values vector will contain: [a, b, b, c]. Read
    together, element by element, they give the map couples.

    :   **Returns:**: a vector of vectors, with the first vector being the keys vector and the second being the values vector.

---


### getKeyValuesPairsSize

```
public int getKeyValuesPairsSize()
```

:   Get the number of unique key-value pairs in the map. No key can be null.
    At most one of the keys can be an empty string. The values can be empty
    strings.

    :   **Returns:**: the number of unique key-value pairs in the map

---


### getNonEmptyKeys

```
public java.util.Set<T> getNonEmptyKeys()
```

:   Get the set of unique, non-empty-string keys for this map.

    :   **Returns:**: the set of unique, non-empty-string keys for this map

---


### getNonEmptyKeyValuesPairsSize

```
public int getNonEmptyKeyValuesPairsSize()
```

:   Get the number of unique, non-empty key-value pairs in the map. No key
    can be null. Empty string keys will not be included, nor will empty
    string values.

    :   **Returns:**: the number of unique key-value pairs in the map

---


### getNonEmptyValues

```
public java.util.Set<U> getNonEmptyValues()
```

:   Get the set of unique, non-empty-string values for this map.

    :   **Returns:**: the set of unique, non-empty-string values for this map

---


### mapIsNullOrEmpty

```
public boolean mapIsNullOrEmpty()
```

:   public void initialise(Vector keys, Vector values) { this.setMap();
    this.generateMap(keys, values);
    }

---


### removeValue

```
public void removeValue(T key,
                        U value)
```

:   Remove a value from the key's list of values

    :   **Parameters:**: `key` -: `value` -

---


### removeValue

```
public void removeValue(U value)
```

:   Remove a value from \*all\* the key's list of values

    :   **Parameters:**: `key` -: `value` -

---


### toCSV

```
public java.lang.String toCSV()
```

:   Get a list of comma separated [key, value] couples.

    :   **Returns:**: a list of comma separated [key, value] couples.

---


### toString

```
public java.lang.String toString()
```

:   **Overrides:**: `toString` in class `java.util.AbstractMap<T,java.util.Set<U>>`

---


### clear

```
public void clear()
```

:   **Specified by:**: `clear` in interface `java.util.Map<T,java.util.Set<U>>` **Overrides:**: `clear` in class `java.util.TreeMap<T,java.util.Set<U>>`

---


### isEmpty

```
public boolean isEmpty()
```

:   **Specified by:**: `isEmpty` in interface `java.util.Map<T,java.util.Set<U>>` **Overrides:**: `isEmpty` in class `java.util.AbstractMap<T,java.util.Set<U>>`

---


### getValues

```
public java.util.Set<U> getValues()
```

:   Get the set of unique values for this map. (No value can be null if type
    is String, or other class whose comparator does not allow null!). At most
    one value can be an empty string.

    :   **Returns:**: the set of unique values for this map

---


### addAll

```
public void addAll(java.util.Map<T,U> map)
```

:   **Parameters:**: `instanceNamesToAttributesStringMap` -

---


### invertKeysAndValues

```
public static OneToManyMap<java.lang.String,java.lang.String> invertKeysAndValues(OneToManyMap<java.lang.String,java.lang.String> map)
```

---


### removeValues

```
public void removeValues(java.util.Collection<U> values)
```

:   **Parameters:**: `queries` -


---


|  |  |  |
| --- | --- | --- |
| **Overview** \| **Package** \| **Class** \| **Use** \| **Tree** \| **Deprecated** \| **Index** \| **Help** | |  |
| **PREV CLASS**   **NEXT CLASS** | **FRAMES**    **NO FRAMES**     **All Classes** |  |
| SUMMARY: NESTED \| FIELD \| CONSTR \| METHOD | DETAIL: FIELD \| CONSTR \| METHOD |  |


---
